# Predicting Distant Metastasis in Young-Onset Colorectal Cancer After Surgery: A Retrospective Study

**DOI:** 10.3389/fonc.2022.804038

**Published:** 2022-02-24

**Authors:** Jie Cheng, Yao-Jia Lao, Qian Wang, Kai Huang, Juan-Li Mou, Jia-Hui Feng, Fan Hu, Meng-Lu Lin, Jun Lin

**Affiliations:** ^1^ Department of Gastroenterology/Hepatology, Zhongnan Hospital of Wuhan University, Wuhan, China; ^2^ The Hubei Clinical Center & Key Laboratory of Intestinal & Colorectal Diseases, Wuhan, China

**Keywords:** colorectal cancer, young-onset, distant metastasis, risk factor, nomogram

## Abstract

**Background:**

Although overall colorectal cancer (CRC) cases have been declining worldwide, there has been an increase in the incidence of the CRC among individuals younger than 50 years old, which is associated with distant metastasis (DM) and poor prognosis.

**Methods:**

Young-onset CRC patients’ postoperative data were collected from the Surveillance, Epidemiology, and End Results (SEER) database between January 2010 and December 2015. Data from the SEER database were divided into early stage and advanced stage according to whether chemoradiotherapy was recommended in the guidelines. Independent risk factors for DM were explored by using univariate and multivariate logistic regression separately. A predictive model was established and presented as nomogram in the training set of advanced stage. The model was internally verified in testing set and externally validated in a cohort of 145 patients from Zhongnan Hospital of Wuhan University. The accuracy, reliability, and clinical application value were assessed using the receiver operating characteristic curve (ROC), the area under the curve (AUC), calibration curve, and decision curve analysis (DCA), respectively. Different risk subgroups of DM were classified according to the scores of the nomogram in the training set of advanced stage.

**Results:**

A total of 5,584 patients were eligible and enrolled in our study in which 1,277 were in early stage and 4,307 in advanced stage. Preoperative CEA positive was found to be an independent predictor of DM in early stage. Multivariate logistic regression analysis showed that tumor size, degree of differentiation, T stage, N stage, preoperative CEA, and whether radiation or chemotherapy performed were independent risk factors for DM (all, *p* < 0.05) in advanced stage. Great accuracies were achieved in our nomogram with AUC of 0.801 in training set, 0.811 in testing set, and 0.791 in the validation cohort, respectively. Calibration curves and DCA in internal validation and external validation both showed good stability and clinical utility values.

**Conclusions:**

Preoperative CEA positive was a significant predictor of DM for young-onset CRC patients. A novel nomogram containing clinical and pathological features was established for predicting DM of advanced CRC in patients younger than 50 years old. This tool may serve as an early alert for clinicians to DM and make better clinical treatment regimens.

## Introduction

Although regular screening and progressive therapeutics are shown to be effective in preventing mortality rate in colorectal cancer (CRC), CRC remains the third leading cause of cancer death worldwide, with more than 935,000 deaths each year (1). The patients younger than the age of 50 years old are deemed as young-onset CRC. It is also worth mentioning that the incidence and mortality of young-onset CRC have significantly increased from 1995 to 2016 in the USA (2). The incidence rate of young-onset CRC has increased from 8.6 per 100,000 people in 1992 to 13.1 per 100,000 people in 2016 in the USA, equally accompanied with high mortality (3, 4). Young-onset CRC is more prone to distant metastasis (DM) and microsatellite instability compared with the elderly, which are associated with adverse outcomes (5). However, the characteristics and clues of DM in young-onset CRC are still insufficient. It is of great importance to assess and predict DM status accurately for treatment decision making and prognostic evaluation in young-onset CRC.

In fact, DM is now the predominant reason for treatment failure with malignant tumor. Approximately 20% of patients with CRC are diagnosed in more advanced stages with synchronous DM (6). Liver and lung are the most common metastases sites with diverse patterns, which may increase the level of treatment difficulty of CRC (7). Hence, detecting DM as early as possible has important clinical application value. Young-onset CRC contributes significantly to the global burden of disease. In the update of clinical practice of young adults with CRC, it is mentioned that young patients with CRC symptoms should have gradually attracted attention (8). With the increasing emphasis put on young-onset, there is still no clinical model to predict DM in young-onset CRC patients. Therefore, we aim to explore the risk factors of DM in young-onset CRC patients after surgery and try to establish a model for predicting DM in this group of patients.

## Materials and Methods

### Participants of Inclusion

Data in this retrospective cohort study were obtained from the Surveillance, Epidemiology, and End Results (SEER) database. The software of SEER*Stata 8.3.9.2 (http://seer.cancer.gov/seerstat/) was utilized to filter and download data of CRC diagnosed during 2010 to 2015. Firstly, CRC patients in the age group of 20 to 49 years old were selected for the study. We excluded the following patients (1) patients without the TNM staging data which was based on the 7th American Joint Committee on Cancer (AJCC) or defined as in situ cancer; (2) patients diagnosed as “autopsy only” or “death certificate only”; (3) patients with 2 or more primary tumors; (4) patients with appendiceal tumors or gastrointestinal stromal tumors; (5) patients with incomplete DM information; (6) patients without surgery performed. We analyzed the information of age, gender, race, marriage, tumor location, tumor size, histology, degree of differentiation, TNM stage, radiation performed, chemotherapy performed, carcinoembryonic antigen (CEA) pretreatment, and follow-up time of every young-onset CRC patient. Overall survival (OS) and cancer-specific survival (CSS) were also recorded. Since patients of pT1-2N0 were not recommended chemoradiotherapy routinely postoperatively on the basis of the clinical practice guidelines on colorectal cancer (9, 10), all the enrolled patients were stratified into early stage and advanced stage based on pT1-2N0. The early stage young-onset CRC was defined as pT1-2N0 without consideration of adjuvant treatment factors, while all other stages except pT1-2N0 were proposed as advanced stage. Patients in the advanced stage were randomly separated into training set and testing set with a ratio of 3:1. A small cohort of advanced stage was collected from Zhongnan Hospital of Wuhan University to validate model externally. This study was conducted in line with the Declaration of Helsinki and approved by the Ethics Committee of Zhongnan Hospital of Wuhan University (number: 2020074).

### Risk Factors Exploration and Nomogram Establishment

Risk factors of DM in early and advanced stage were explored, respectively. Patients in the derivation cohort were assigned into two groups according to whether DM occurred (M0 and M1). We used the Kaplan-Meier method to evaluate the OS and CSS in different groups. In order to investigate independent risk factors of young-onset CRC patients, the Chi-square test and univariate and multivariate logistic regression were adopted. A nomogram prediction model was established based on the results of multivariate logistic regression analysis in the training set and was internally validated in the testing set and externally validated in the validation test. The predictive accuracy of the nomogram was assessed by using receiver operating characteristic curve (ROC) analysis and calculating the area under the curve (AUC). Calibration curves were used to compare the consistency of the predicted and actual probabilities of the nomogram. Meanwhile, we applied decision curve (DCA) analysis and clinical impact curve (CIC) to assess the clinical effectiveness of the model by calculating the net benefits under each risk threshold probability. We computed all the risk scores according to the nomogram and divided them into low, medium, and high risk separately. Prognosis was also evaluated by the risk stratification eventually.

### Statistical Analysis

All statistical analyses were performed using SPSS version 25.0 and the R version 3.6.1. Continuous variables were reported as median with interquartile range (IQR), while categorical variables were reported as number with proportions. Chi-square test or Fisher’s exact test was performed for the comparison of variables of majority pathological features. Kaplan-Meier curves and log-rank test were used to analyze the survival in patients with or without DM. For variables with *p*-value <0.05 in the univariate logistic regression analysis, multivariate regression analysis using a forward stepwise method was adopted to identify independent risk factors of DM. Nomogram, calibration curves, ROC, DCA, and CIC were performed or plotted using R version 3.6.1 ultimately. Two-sided *p*-values <0.05 were considered statistical significance.

## Results

### Basic Characteristics of Patients

**Figure 1:**
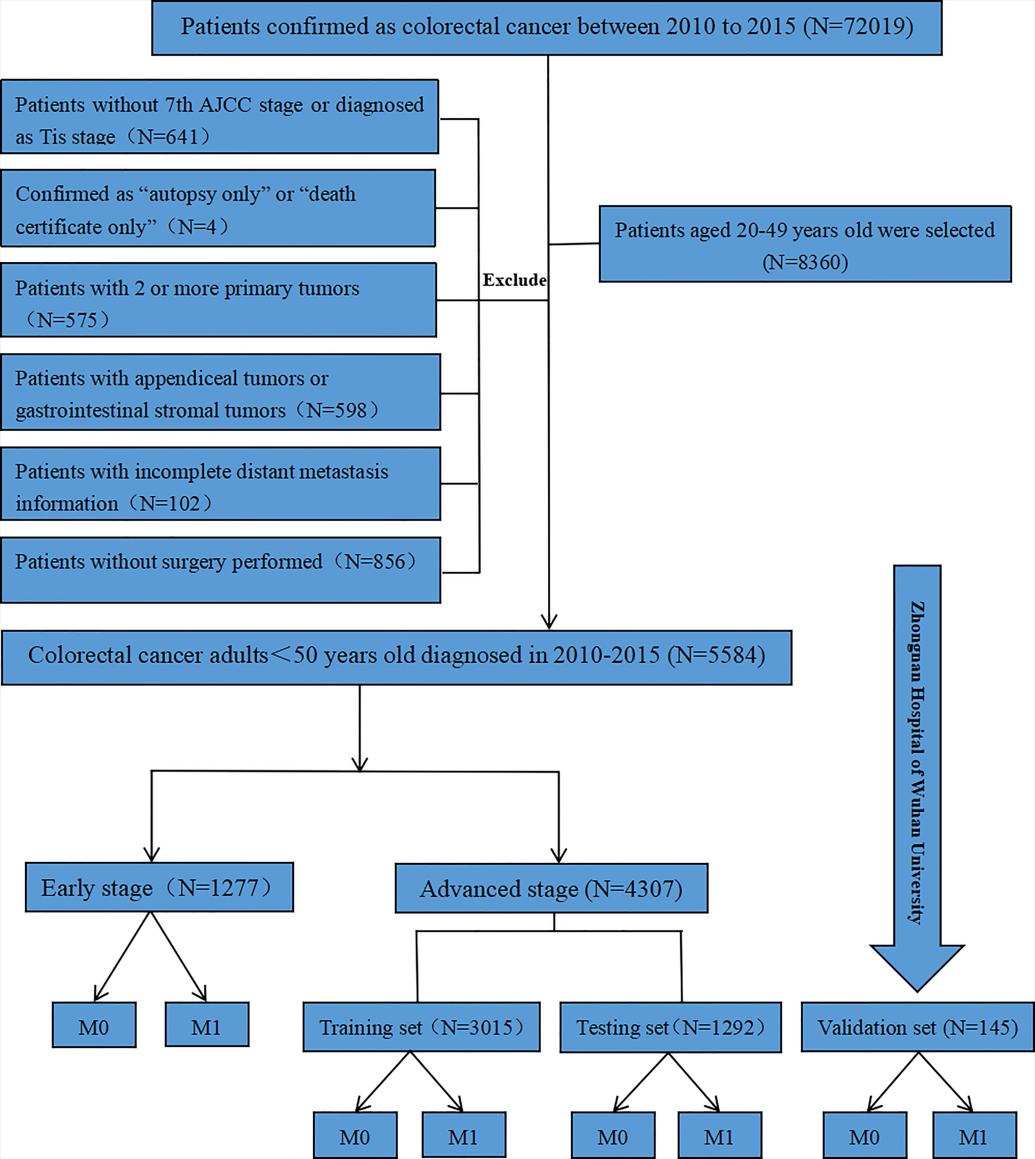
Flow chart of participants inclusion. M1, distant metastasis; M0, no distant metastasis.

**Figure 2:**
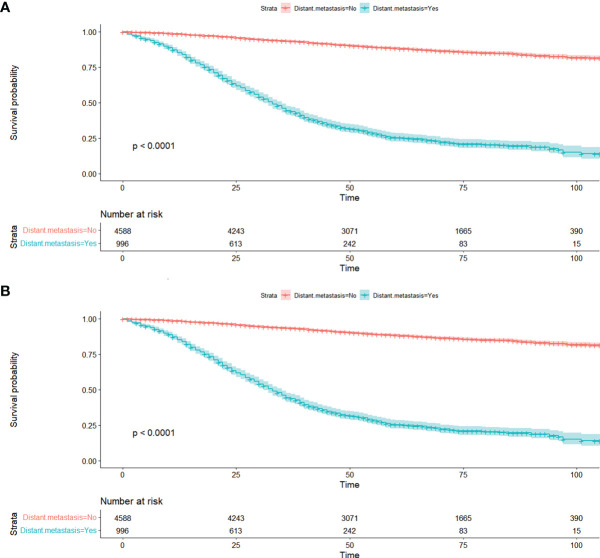
Kaplan-Meier curves of distant metastasis for OS **(A)** and CSS **(B)** in all patients from SEER database. OS, overall survival; CSS, cancer-specific survival; SEER, Surveillance, Epidemiology, and End Results database.

A total of 5,584 young-onset CRC patients who underwent surgical resection were included in this study from SEER database, among whom 996 patients developed DM with only 25 in the early stage group and 971 in the advanced stage group. In DM patients, the most common site for metastasis organ was liver (68.0%, 677/996), followed by lung (14.7%, 146/996), bone (2.3%, 23/996), and brain (0.6%, 6/996). The basic characteristics of all patients from SEER database are presented in **Table 1**. Among patients included, 1,277 were classified into the early stage group and 4,307 into the advanced stage group. There were obvious statistical differences between the two groups in terms of gender, race, marriage, tumor location, tumor size, histology, degree of differentiation, T stage, and N stage, whether radiation was performed, whether chemotherapy was performed, CEA pretreatment, and whether DM occurred (*p* < 0.05), as shown in **Table 2**. In the group of advanced stage, the data were randomly classified into a training set of 3,015 individuals and a testing set of 1,292 individuals. No significant difference was observed in basic demographic and pathological characteristics between the two sets (**Table 3**). A cohort of 145 young-onset CRC patients in advanced stage from Zhongnan Hospital of Wuhan University was defined as the validation set, among whom 31 patients developed DM. A flow chart of inclusion of patients was shown in **Figure 1**. The Kaplan-Meier curves revealed that patients with DM had a worse prognosis than patients without DM (**Figure 2**).

**Table 1:** Clinicopathological characteristics of young-onset colorectal cancer by metastatic site in all including patients from the SEER database.

Variables	ALL		*p*	Distant metastatic site			
	M1 (*N* = 996)	M0 (*N* = 4,588)		Liver (*N* = 677)	Lung (*N* = 146)	Bone (*N* = 23)	Brain (*N* = 6)
**Age**							
20–29	41 (4.1)	220 (4.8)	0.608	25 (3.7)	3 (2.1)	0 (0.0)	0 (0.0)
30–39	204 (20.5)	958 (20.9)		127 (18.8)	28 (19.2)	11 (47.8)	1 (16.7)
40–49	751 (75.4)	3,410 (74.3)		525 (77.5)	115 (78.8)	12 (52.2)	5 (83.3)
**Gender**							
Female	466 (46.8)	2,135 (46.5)	0.885	309 (45.6)	73 (50.0)	9 (39.1)	4 (66.7)
Male	530 (53.2)	2,453 (53.5)		368 (54.4)	73 (50.0)	14 (60.9)	2 (33.3)
**Race**							
White	715 (71.8)	3,233 (70.5)	0.049	479 (70.8)	98 (67.1)	16 (69.6)	5 (83.3)
Black	160 (16.1)	660 (14.4)		120 (17.7)	27 (18.5)	1 (4.3)	1 (16.7)
Other	116 (11.6)	648 (14.1)		76 (11.2)	20 (13.7)	6 (26.1)	0 (0.0)
Unknown	5 (0.5)	47 (1.0)		2 (0.3)	1 (0.7)	0 (0.0)	0 (0.0)
**Marriage**							
Single	398 (40.0)	1,715 (37.4)	0.160	254 (37.5)	57 (39.0)	9 (31.9)	1 (16.7)
Married	554 (55.6)	2,619 (57.1)		394 (58.2)	83 (56.8)	13 (56.5)	4 (66.7)
Unknown	44 (4.4)	254 (5.5)		29 (4.3)	6 (4.1)	1 (4.3)	1 (16.7)
**Tumor location**							
Left colon	507 (50.9)	1,885 (41.1)	<0.001	356 (52.6)	68 (46.6)	11 (47.8)	1 (16.7)
Right colon	285 (28.6)	1,122 (24.5)		188 (27.8)	32 (21.9)	2 (8.7)	2 (33.3)
Rectum	173 (17.4)	1,533 (33.4)		114 (16.8)	40 (27.4)	8 (34.8)	3 (50.0)
Unknown	31 (3.1)	48 (1.0)		19 (2.8)	6 (4.1)	2 (8.7)	0 (0.0)
**Tumor size**							
0–10 mm	7 (0.7)	483 (10.5)	<0.001	6 (0.9)	0 (0.0)	1 (4.3)	0 (0.0)
11–20 mm	34 (3.4)	308 (6.7)		25 (3.7)	2 (1.4)	1 (4.3)	0 (0.0)
21–30 mm	95 (9.5)	505 (11.0)		65 (9.6)	19 (13.0)	2 (8.7)	0 (0.0)
31–40 mm	164 (16.5)	685 (14.9)		116 (17.1)	21 (14.4)	2 (8.7)	2 (33.3)
41–50 mm	206 (20.7)	722 (15.7)		149 (22.0)	30 (20.5)	4 (17.7)	1 (16.7)
50+ mm	412 (41.4)	1,441 (31.4)		271 (40.0)	60 (41.1)	7 (30.4)	3 (50.0)
Unknown	78 (7.8)	444 (9.7)		45 (6.6)	14 (9.6)	6 (26.1)	0 (0.0)
**Histology**							
Adenocarcinoma	862 (86.5)	3,902 (85.0)	0.008	617 (91.1)	140 (95.9)	20 (87.0)	6 (100.0)
Mucinous carcinoma	76 (7.6)	294 (6.4)		31 (4.6)	4 (2.7)	1 (4.3)	0 (0.0)
Unknown/other	58 (5.8)	392 (8.5)		29 (4.3)	2 (1.4)	2 (8.7)	0 (0.0)
**Degree of differentiation**							
Well differentiated	36 (3.6)	444 (9.7)	<0.001	25 (3.7)	5 (3.4)	0 (0.0)	0 (0.0)
Moderately differentiated	600 (60.2)	3,097 (67.5)		440 (65.0)	94 (64.4)	9 (39.1)	3 (50.0)
Poorly differentiated	229 (23.0)	551 (12.0)		136 (20.1)	24 (16.4)	5 (21.7)	3 (50.0)
Undifferentiated	57 (5.7)	107 (2.3)		33 (4.9)	12 (8.2)	4 (17.4)	0 (0.0)
Unknown	74 (7.4)	389 (8.5)		43 (6.4)	11 (7.5)	5 (21.7)	0 (0.0)
**T stage**							
T1	31 (3.1)	963 (21.0)	<0.001	24 (3.5)	1 (0.7)	2 (8.7)	0 (0.0)
T2	27 (2.7)	550 (12.0)		25 (3.7)	3 (2.1)	1 (4.3)	0 (0.0)
T3	479 (48.1)	2,437 (53.1)		360 (53.2)	76 (52.1)	8 (34.8)	5 (83.3)
T4	429 (43.1)	624 (13.6)		245 (36.2)	59 (40.4)	12 (52.2)	1 (16.7)
Tx/unknown	30 (3.0)	14 (0.3)		23 (3.4)	7 (4.8)	0 (0.0)	0 (0.0)
**N stage**							
N0	143 (14.4)	2,415 (52.6)	<0.001^a^	97 (14.3)	28 (19.2)	1 (4.3)	2 (33.3)
N1	402 (40.4)	1,389 (30.3)		277 (40.9)	63 (43.2)	11 (47.8)	0 (0.0)
N2	442 (44.4)	784 (17.1)		297 (43.7)	53 (36.3)	10 (43.5)	4 (66.7)
Nx	9 (0.9)	0 (0.0)		6 (0.9)	2 (1.4)	1 (4.3)	0 (0.0)
**Radiation performed**							
No	826 (82.9)	3,422 (74.6)	<0.001	572 (84.5)	110 (75.3)	14 (60.9)	1 (16.7)
Yes	170 (17.1)	1,166 (25.4)		105 (15.5)	36 (24.7)	9 (39.1)	5 (83.3)
**Chemotherapy performed**							
No	88 (8.8)	1,934 (42.2)	<0.001	61 (9.0)	17 (11.6)	2 (8.7)	0 (0.0)
Yes	908 (91.2)	2,654 (57.8)		616 (91.0)	129 (88.4)	21 (91.3)	6 (100.0)
**CEA pretreatment**							
CEA negative/normal	198 (19.9)	1,814 (39.5)	<0.001^a^	113 (16.7)	23 (15.8)	4 (17.4)	3 (50.0)
CEA positive/elevated	513 (51.5)	857 (18.7)		381 (56.3)	82 (56.2)	11 (47.8)	2 (33.3)
Borderline	4 (0.4)	6 (0.1)		1 (0.1)	0 (0.0)	0 (0.0)	0 (0.0)
Unknown	281 (28.2)	1,911 (41.7)		182 (26.9)	41 (28.1)	8 (34.8)	1 (16.7)

SEER, Surveillance, Epidemiology, and End Results database; M0, distant metastasis; M1, no distant metastasis.

a Number: adopting Fisher’s exact test.

**Table 2:** Clinicopathological characteristics of young-onset colorectal cancer between early stage and advanced stage.

Variables	Early Stage (*N* = 1,277)	Advanced Stage (*N* = 4,307)	*p*
**Age**			
20–29	62 (4.9)	199 (4.6)	0.864
30–39	260 (20.4)	902 (20.9)	
40–49	955 (74.8)	3,206 (74.4)	
**Gender**			
Female	633 (49.6)	1,968 (45.7)	0.015
Male	644 (50.4)	2,339 (54.3)	
**Race**			
White	874 (68.4)	3,074 (71.4)	<0.001
Black	203 (15.9)	617 (14.3)	
Other	174 (13.6)	590 (13.7)	
Unknown	26 (2.0)	26 (0.6)	
**Marriage**			
Single	453 (35.5)	1,660 (38.5)	<0.001
Married	707 (55.4)	2,466 (57.3)	
Unknown	117 (9.2)	181 (4.2)	
**Tumor location**			
Left colon	481 (37.7)	1,911 (44.4)	<0.001
Right colon	202 (15.8)	1,205 (28.0)	
Rectum	584 (45.7)	1,122 (26.1)	
Unknown	10 (0.8)	69 (1.6)	
**Tumor size**			
0–10 mm	427 (33.4)	63 (1.5)	<0.001
11–20 mm	162 (12.7)	180 (4.2)	
21–30 mm	149 (11.7)	451 (10.5)	
31–40 mm	118 (9.2)	731 (17.0)	
41–50 mm	84 (6.6)	844 (19.6)	
50+ mm	74 (5.8)	1,779 (41.3)	
Unknown	263 (20.6)	259 (6.0)	
**Histology**			
Adenocarcinoma	950 (74.4)	3,814 (88.6)	<0.001
Mucinous carcinoma	26 (2.0)	344 (8.0)	
Unknown/other	301 (23.6)	149 (3.5)	
**Degree of differentiation**			
Well differentiated	258 (20.2)	222 (5.2)	<0.001
Moderately differentiated	724 (56.7)	2,973 (69.0)	
Poorly differentiated	69 (5.4)	711 (16.5)	
Undifferentiated	8 (0.6)	156 (3.6)	
Unknown	218 (17.1)	245 (5.7)	
**T stage**			
T1	886 (69.4)	108 (2.5)	<0.001^a^
T2	391 (30.6)	186 (4.3)	
T3	0 (0.0)	2,916 (67.7)	
T4	0 (0.0)	1,053 (24.4)	
Tx/Unknown	0 (0.0)	44 (1.0)	
**N stage**			
N0	1,277 (100.0)	1,281 (29.7)	<0.001^a^
N1	0 (0.0)	1,791 (41.6)	
N2	0 (0.0)	1,226 (28.5)	
Nx	0 (0.0)	9 (0.2)	
**Radiation performed**			
No	1,186 (92.9)	3,062 (71.1)	<0.001
Yes	91 (7.1)	1,245 (28.9)	
**Chemotherapy performed**			
No	1,160 (90.8)	862 (20.0)	<0.001
Yes	117 (9.2)	3,445 (80.0)	
**CEA pretreatment**			
CEA negative/normal	380 (29.8)	1,632 (37.9)	<0.001^a^
CEA positive/elevated	61 (4.8)	1,309 (30.4)	
Borderline	1 (0.1)	9 (0.2)	
Unknown	835 (65.4)	1,357 (31.5)	
**Distant metastasis**			
No	1,252 (98.0)	3,336 (77.5)	<0.001
Yes	25 (2.0)	971 (22.5)	
**Follow-up time**	67 (48–87)	53 (37–79)	<0.001

Early stage: pT1-2N0; advanced stage: other stages except pT1-2N0.

a Number: adopting Fisher’s exact test.

**Table 3:** Clinicopathological characteristics of young-onset colorectal cancer in training set and testing set in advanced stage group.

Variables	Training Set (*N* = 3,015)	Testing Set (*N* = 1,292)	*p*
**Age**			
20–29	135 (4.5)	64 (5.0)	0.737
30–39	637 (21.1)	265 (20.5)	
40–49	2,243 (74.4)	963 (74.5)	
**Gender**			
Female	1,360 (45.1)	608 (47.1)	0.239
Male	1,655 (54.9)	684 (52.9)	
**Race**			
White	2,143 (71.1)	931 (72.1)	0.921
Black	435 (14.4)	182 (14.1)	
Other	419 (13.9)	171 (13.2)	
Unknown	18 (0.6)	8 (0.6)	
**Marriage**			
Single	1,152 (38.2)	508 (39.3)	0.789
Married	1,736 (57.6)	730 (56.5)	
Unknown	127 (4.2)	54 (4.2)	
**Tumor location**			
Left colon	1,337 (44.3)	574 (44.4)	0.617
Right colon	855 (28.4)	350 (27.1)	
Rectum	772 (25.6)	350 (27.1)	
Unknown	51 (1.7)	18 (1.4)	
**Tumor size**			
0–10 mm	46 (1.5)	17 (1.3)	0.059
11–20 mm	115 (3.8)	65 (5.0)	
21–30 mm	324 (10.7)	127 (9.8)	
31–40 mm	496 (16.5)	235 (18.2)	
41–50 mm	577 (19.1)	267 (20.7)	
50+ mm	1,284 (42.6)	495 (38.3)	
Unknown	173 (5.7)	86 (6.7)	
**Histology**			
Adenocarcinoma	2,660 (88.2)	1,154 (89.3)	0.586
Mucinous carcinoma	248 (8.2)	96 (7.4)	
Unknown/other	107 (3.5)	42 (3.3)	
**Degree of differentiation**			
Well differentiated	146 (4.8)	76 (5.9)	0.282
Moderately differentiated	2,104 (69.8)	869 (67.3)	
Poorly differentiated	481 (16.0)	230 (17.8)	
Undifferentiated	113 (3.7)	43 (3.3)	
Unknown	171 (5.7)	74 (5.7)	
**T stage**			
T1	65 (2.2)	43 (3.3)	0.077
T2	126 (4.2)	60 (4.6)	
T3	2,068 (68.6)	848 (65.6)	
T4	729 (24.2)	324 (25.1)	
Tx/Unknown	27 (0.9)	17 (1.3)	
**N stage**			
N0	907 (30.1)	374 (28.9)	0.405^a^
N1	1,229 (40.8)	562 (43.5)	
N2	872 (28.9)	354 (27.4)	
Nx	7 (0.2)	2 (0.2)	
**Radiation performed**			
No	2,142 (71.0)	920 (71.2)	0.914
Yes	873 (29.0)	372 (28.8)	
**Chemotherapy performed**			
No	605 (20.1)	257 (19.9)	0.896
Yes	2,410 (79.9)	1,035 (80.1)	
**CEA pretreatment**			
CEA negative/normal	1,150 (38.1)	482 (37.3)	0.934^a^
CEA positive/elevated	913 (30.3)	396 (30.7)	
Borderline	6 (0.2)	3 (0.2)	
Unknown	946 (31.4)	411 (31.8)	
**Distant metastasis**			
No	2,342 (77.7)	994 (76.9)	0.593
Yes	673 (22.3)	298 (23.1)	
**Follow-up time**	53 (37–79)	54 (37–79)	0.900

a Number: adopting Fisher’s exact test.

### Independent Risk Factors of DM in Early Stage Young-Onset CRC

As presented in **Table 4**, only 25 patients developed DM in the early stage group. Results of Chi-square tests or Fisher’s exact test indicated that pretreatment CEA (*p* < 0.001) and tumor size (*p* = 0.022) were associated with DM. Univariate logistic analysis showed that tumor size was not the independent risk factor of DM (*p* = 0.058) while pretreatment CEA was an independent risk factor of DM in early stage. Comparing with pretreatment CEA negative, patients with pretreatment CEA positive were more likely to develop DM (OR = 30.776, 95% CI = 8.390–112.889, *p* < 0.001).

**Table 4:** Risk factors associated with distant metastasis of young-onset colorectal cancer in early stage group.

Variables	Early Stage		*p*
	M1 (*N* = 25)	M0 (*N* = 1,252)	
**Age**			
20–29	0 (0.0)	62 (5.0)	0.251^a^
30–39	8 (32.0)	252 (20.1)	
40–49	17 (68.0)	938 (74.9)	
**Gender**			
Female	9 (36.0)	624 (49.8)	0.171
Male	16 (64.0)	628 (50.2)	
**Race**			
White	15 (60.0)	859 (68.6)	0.417^a^
Black	7 (28.0)	196 (15.7)	
Other	3 (12.0)	171 (13.7)	
Unknown	0 (0.0)	26 (2.1)	
**Marriage**			
Single	11 (44.0)	442 (35.3)	0.558^a^
Married	13 (52.0)	694 (55.4)	
Unknown	1 (4.0)	116 (9.3)	
**Tumor location**			
Left colon	10 (40.0)	471 (37.6)	0.291^a^
Right colon	7 (28.0)	195 (15.6)	
Rectum	8 (32.0)	576 (46.0)	
Unknown	0 (0.0)	10 (0.8)	
**Tumor size**			
0–10 mm	2 (8.0)	425 (33.9)	0.022^a^
11–20 mm	4 (16.0)	158 (12.6)	
21–30 mm	4 (16.0)	145 (11.6)	
31–40 mm	6 (24.0)	112 (8.9)	
41–50 mm	1 (4.0)	83 (6.6)	
50+ mm	2 (8.0)	72 (5.8)	
Unknown	6 (24.0)	257 (20.5)	
**Histology**			
Adenocarcinoma	22 (88.0)	928 (74.1)	0.334^a^
Mucinous carcinoma	0 (0.0)	26 (2.1)	
Unknown/other	3 (12.0)	298 (23.8)	
**Degree of differentiation**			
Well differentiated	2 (8.0)	256 (20.4)	0.400^a^
Moderately differentiated	15 (60.0)	709 (56.6)	
Poorly differentiated	2 (8.0)	67 (5.4)	
Undifferentiated	0 (0.0)	8 (0.6)	
Unknown	6 (24.0)	212 (16.9)	
**T stage**			
T1	15 (60.0)	871 (69.6)	0.304
T2	10 (40.0)	381 (30.4)	
**CEA pretreatment**			
CEA negative/normal	3 (12.0)	377 (30.1)	<0.001^a^
CEA positive/elevated	12 (48.0)	49 (3.9)	
Borderline	0 (0.0)	1 (0.1)	
Unknown	10 (40.0)	825 (65.9)	

M0, distant metastasis; M1, no distant metastasis.

a Number: adopting Fisher’s exact test.

### Independent Risk Factors of DM in Advanced Stage Young-Onset CRC

In the advanced stage group, results of Chi-square tests or Fisher’s exact test demonstrated that tumor size, location, histology, degree of differentiation, T stage, N stage, radiation performed, chemotherapy performed, and CEA pretreatment were associated with DM (**Table 5**), which were then included in the univariate and multivariate logistic regression analyses. After adjustment for all other risk factors, multivariate logistic regression analysis indicated that patients with the tumor size of 41–50 mm (OR = 4.267, 95% CI = 1.128–16.135, *p* = 0.033) were easier to develop DM when compared with the tumor size of 0–10 mm. Patients with undifferentiated carcinoma had higher risk of DM than those with well differentiated (OR = 2.030, 95% CI = 1.025–4.023, 0.042). Patients with T4 stage (OR = 7.111, 95% CI = 2.667–18.962, *p* <0.001) and N2 stage (OR = 3.970, 95% CI = 2.948–5.345, *p* <0.001) were easier to had DM than those with T1 and N0, respectively. Interestingly, treatment with a combination of surgery and radiotherapy (OR = 0.342, 95% CI = 0.265–0.440, *p* <0.001) were a protective DM factor for young-onset CRC in advanced stage. Surgery combined with chemotherapy was an independent risk factor (OR = 2.250, 95% CI = 1.643–3.081, *p* <0.001). CEA positive (OR = 4.027, 95% CI = 3.160–5.131, *p* <0.001) before treatment was more prone to increase the likelihood of DM than CEA negative (**Table 6**).

**Table 5:** Risk factors associated with distant metastasis of young-onset colorectal cancer in advanced stage group of training set.

Variables	Advanced Stage		*p*
	M1 (*N* = 673)	M0 (*N* = 2,342)	
**Age**			
20–29	29 (4.3)	106 (4.5)	0.939
30–39	140 (20.8)	497 (21.2)	
40–49	504 (74.9)	1,739 (74.3)	
**Gender**			
Female	305 (45.3)	1,055 (45.0)	0.900
Male	368 (54.7)	1,287 (55.0)	
**Race**			
White	473 (70.3)	1,670 (71.3)	0.106^a^
Black	114 (16.9)	321 (13.7)	
Other	84 (12.5)	335 (14.3)	
Unknown	2 (0.3)	16 (0.7)	
**Marriage**			
Single	269 (40.0)	883 (37.7)	0.537
Married	375 (55.7)	1,361 (58.1)	
Unknown	29 (4.3)	98 (4.2)	
**Tumor location**			
Left colon	347 (51.6)	990 (42.3)	<0.001
Right colon	191 (28.4)	664 (28.4)	
Rectum	113 (16.8)	659 (28.1)	
Unknown	22 (3.3)	29 (1.2)	
**Tumor size**			
0–10 mm	4 (0.6)	42 (1.8)	0.006
11–20 mm	16 (2.4)	99 (4.2)	
21–30 mm	62 (9.2)	262 (11.2)	
31–40 mm	103 (15.3)	393 (16.8)	
41–50 mm	147 (21.8)	430 (18.4)	
50+ mm	294 (43.7)	990 (42.3)	
Unknown	47 (7.0)	126 (5.4)	
**Histology**			
Adenocarcinoma	581 (86.3)	2,079 (88.8)	0.004
Mucinous carcinoma	54 (8.0)	194 (8.3)	
Unknown/other	38 (5.6)	69 (2.9)	
**Degree of differentiation**			
Well differentiated	19 (2.8)	127 (5.4)	<0.001
Moderately differentiated	422 (62.7)	1,682 (71.8)	
Poorly differentiated	147 (21.8)	334 (14.3)	
Undifferentiated	40 (5.9)	73 (3.1)	
Unknown	45 (6.7)	126 (5.4)	
**T stage**			
T1	7 (1.0)	58 (2.5)	<0.001
T2	11 (1.6)	115 (4.9)	
T3	343 (51.0)	1,725 (73.7)	
T4	295 (43.8)	434 (18.5)	
Tx/Unknown	17 (2.5)	10 (0.4)	
**N stage**			
N0	80 (11.9)	827 (35.3)	<0.001^a^
N1	268 (39.8)	961 (41.0)	
N2	318 (47.3)	554 (23.7)	
Nx	7 (1.0)	0 (0.0)	
**Radiation performed**			
No	557 (82.8)	1,585 (67.7)	<0.001
Yes	116 (17.2)	757 (32.3)	
**Chemotherapy performed**			
No	63 (9.4)	542 (23.1)	<0.001
Yes	610 (90.6)	1,800 (76.9)	
**CEA pretreatment**			
CEA negative/normal	138 (20.5)	1,012 (43.2)	<0.001^a^
CEA positive/elevated	342 (50.8)	571 (24.4)	
Borderline	1 (0.1)	5 (0.2)	
Unknown	192 (28.5)	754 (32.2)	

M0, distant metastasis; M1, no distant metastasis.

a Number: adopting Fisher’s exact test.

**Table 6:** Univariate and multivariate logistic regression analyses of distant metastasis in advanced stage young-onset colorectal cancer from training set.

Variables	Univariate Analysis		Multivariate Analysis	
	OR (95% CI)	*p*	OR (95% CI)	*p*
**Tumor location**		<0.001		0.364
Left colon	Reference		Reference	
Right colon	0.821 (0.670–1.004)	0.055	–	0.207
Rectum	0.489 (0.387–0.618)	<0.001	–	0.917
Unknown	2.164 (1.227–3.818)	0.008	–	0.162
**Tumor size**		0.008		0.049
0–10 mm	Reference		Reference	
11–20 mm	1.697 (0.535–5.379)	0.369	3.599 (0.858–15.086)	0.080
21–30 mm	2.485 (0.859–7.188)	0.093	3.305 (0.859–12.719)	0.082
31–40 mm	2.752 (0.965–7.851)	0.058	3.440 (0.903–13.109)	0.070
41–50 mm	3.590 (1.266–10.181)	0.016	4.267 (1.128–16.135)	0.033
50+ mm	3.118 (1.109–8.768)	0.031	3.099 (0.825–11.637)	0.094
Unknown	3.917 (1.332–11.521)	0.013	4.977 (1.279–19.365)	0.021
**Histology**		0.005		0.253
Adenocarcinoma	Reference		Reference	
Mucinous carcinoma	0.996 (0.727–1.365)	0.980	–	0.155
Unknown/other	1.971 (1.312–2.959)	0.001	–	0.329
**Degree of differentiation**		<0.001		0.020
Well differentiated	Reference		Reference	
Moderately differentiated	1.677 (1.024–2.748)	0.040	1.327 (0.772–2.282)	0.306
Poorly differentiated	2.942 (1.750–4.947)	<0.001	1.688 (0.952–2.992)	0.073
Undifferentiated	3.663 (1.976–6.790)	<0.001	2.030 (1.025–4.023)	0.042
Unknown	2.387 (1.323–4.307)	0.004	2.160 (1.104–4.228)	0.025
**T stage**		<0.001		<0.001
T1	Reference		Reference	
T2	0.793 (0.292–2.152)	0.648	1.274 (0.404–4.015)	0.679
T3	1.648 (0.746–3.640)	0.217	2.820 (1.064–7.472)	0.037
T4	5.632 (2.536–12.510)	<0.001	7.111 (2.667–18.962)	<0.001
Tx/Unknown	14.086 (4.657–42.607)	<0.001	20.955 (5.51–79.698)	<0.001
**N stage**		<0.001		
N0	Reference			
N1	2.883 (2.209–3.763)	<0.001	2.641 (1.967–3.545)	<0.001
N2	5.934 (4.540–7.755)	<0.001	3.970 (2.948–5.345)	<0.001
Nx	–	0.999	–	0.998
**Radiation performed**		<0.001		<0.001
No	Reference		Reference	
Yes	0.436 (0.351–0.542)	<0.001	0.342 (0.265–0.440)	<0.001
**Chemotherapy performed**		<0.001		<0.001
No	Reference		Reference	
Yes	2.916 (2.211–3.844)	<0.001	2.250 (1.643–3.081)	<0.001
**CEA pretreatment**		<0.001		<0.001
CEA negative/normal	Reference			
CEA positive/elevated	4.392 (3.515–5.488)	<0.001	4.027 (3.160–5.131)	<0.001
Borderline	1.467 (0.170–12.646)	0.728	1.246 (0.118–13.150)	0.855
Unknown	1.867 (1.472–2.370)	<0.001	1.677 (1.295–2.172)	<0.001

### Nomogram Establishment and Validation

**Figure 3:**
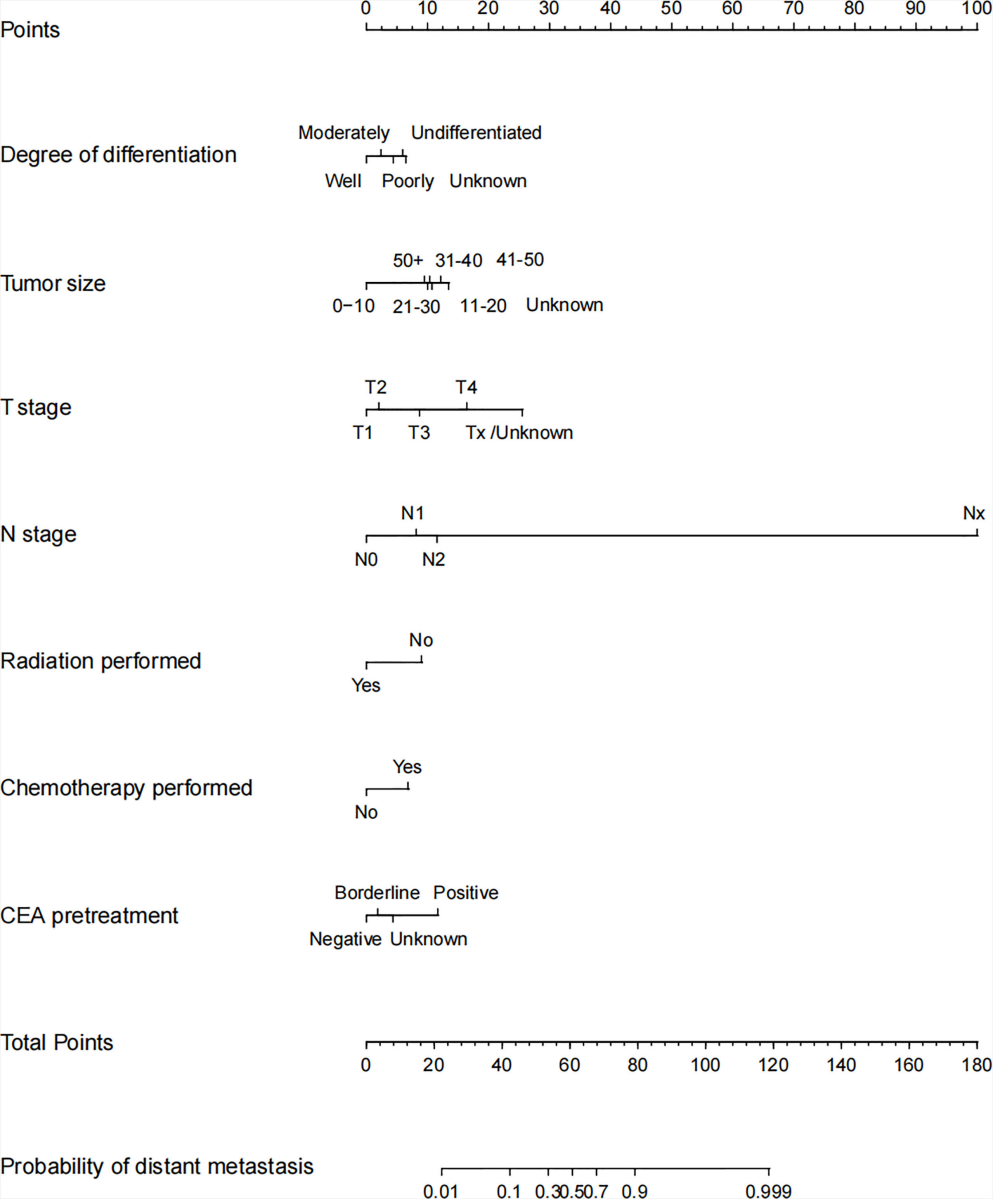
Nomogram for distant metastasis of advanced stage young-onset colorectal cancer patients in training set. To estimate the risk of distant metastasis, the point of each variable was calculated by drawing a straight line from the patient variable value to the axis marked “points.” The total points are converted to the “probability of distant metastasis” on the lowest axis.

Analyses above showed that only the pretreatment CEA positive was an independent risk factor of DM in early stage, while tumor size, degree of differentiation, T stage, N stage, radiation performed, chemotherapy performed, and CEA pretreatment were all independent risk factors of DM in advanced stage. Thus, we established a prediction model of DM in the training set of advanced stage group, which was presented as nomogram to visually illustrate the probabilities of DM (**Figure 3**).

**Figure 4:**
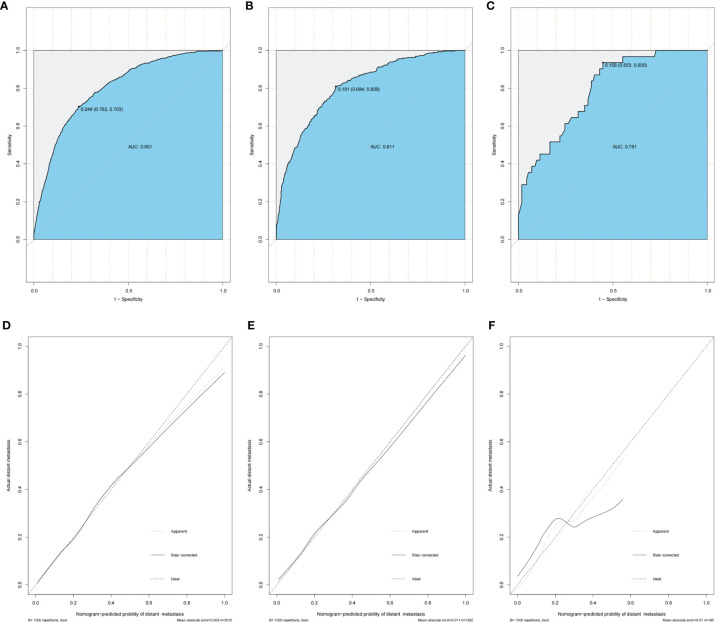
Nomogram ROC curves and calibration curves in training set, testing set, and validation set of advanced stage young-onset colorectal cancer. **(A)** ROC curve of training set for distant metastasis model (AUC = 80.1%). **(B)** ROC curve of testing set for distant metastasis model. The AUC is 81.1%. **(C)** ROC curve of validation set for distant metastasis model (AUC = 79.1%). **(D)** Calibration curve of training set for distant metastasis model. **(E)** Calibration curve of testing set for distant metastasis model. **(F)** Calibration curve of validation set for distant metastasis model. ROC, receiver operating characteristic; AUC, area under the ROC curve.

Accuracy, stability and clinical value of the model were assessed in the training set, testing set, and validation set. The ROC curves were plotted using the pROC package and presented in **Figures 4A–C**, primarily to assess the predictive accuracy of the model. The AUC of our model in training set was 0.801 with sensitivity of 0.762 and specificity of 0.703. Meanwhile, the AUC in the testing set and validation cohort were 0.811 (sensitivity as 0.684 and specificity as 0.809) and 0.791 (sensitivity as 0.553 and specificity as 0.935), respectively, all of which demonstrated good accuracy of prediction. The calibration curves were plotted to evaluate the consistence of the actual probability and the predicted probability of DM in the training set, testing set, and validation set (**Figures 4D–F**), in which no obvious deviations from the reference line were observed, indicating that our model had good consistency in training set, testing set, and validation set.

**Figure 5:**
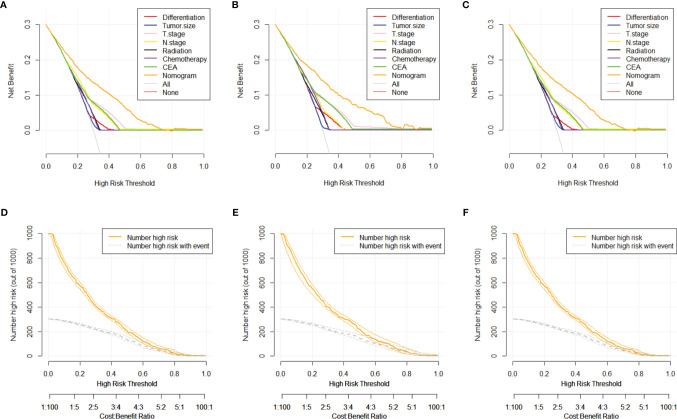
DCA and CIC curves of nomogram for distant metastasis in training set, testing set, and validation set. **(A)** DCA curve of nomogram for distant metastasis in training set. **(B)** DCA curve of nomogram for distant metastasis in testing set. **(C)** DCA curve of nomogram for distant metastasis in validation set. **(D)** CIC curve of nomogram for distant metastasis in training set. **(E)** CIC curve of nomogram for distant metastasis in testing set. **(F)** CIC curve of nomogram for distant metastasis in validation set. DCA, decision curve analysis; CIC, clinical impact curve.

The DCA and CIC analyses were employed for the evaluation of the clinical value of the predictive nomogram. The DCA indicated that the nomogram model revealed higher clinical value than any independent variable, as shown in **Figures 5A–C** with orange lines. The solid line represents the number of people at high risk of DM according to our model, and the dotted line represents the number of people actually metastasized in CIC (**Figures 5D–F**). When the threshold probabilities were 0 to 0.8, the most beneficial clinical value for predicting DM in advanced stage was observed.

### Risk Score in Nomogram

**Figure 6:**
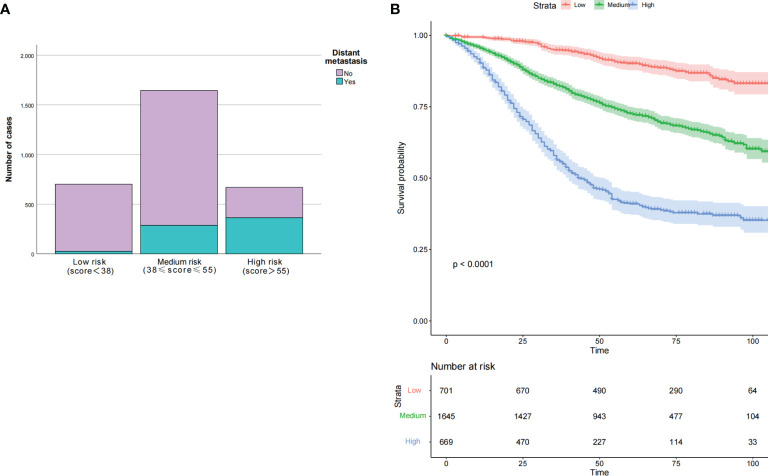
Clinical effects of the risk score in our nomogram in training set. Based on the interquartile range of risk score, our nomogram divided participants into three subgroups. The number of distant metastasis in each subgroup was present in **(A)**, both *p* < 0.001. The OS was also evaluated among the different risk subgroups with *p* < 0.0001 **(B)**. OS, overall survival.

We calculated nomogram scores for all advanced stage patients by using R language. Here, we showed all the score of every clinicopathological variable in our nomogram in **Table 7**. By using the 25th and 75th percentile values of the total risk scores, patients were divided into three groups, low risk (<38), median risk (38–55), and high risk (>55). We found that the number of DM increased along with the increase of risk stratification (*p* < 0.05). The Kaplan-Meier curves were applied to better show the relationship between risk stratification and survival prognosis, which suggested that the higher risk of metastasis, the lower probability of good prognosis or survival in the young-onset patients (**Figure 6**).

**Table 7:** Score of every clinicopathological variable in our nomogram.

Clinicopathological variables	Nomogram score of distant metastasis
**Degree of differentiation**	
Well differentiated	0
Moderately differentiated	2
Poorly differentiated	4
Undifferentiated	6
Unknown	6
**Tumor size**	
0–10 mm	0
11–20 mm	11
21–30 mm	10
31–40 mm	10
41–50 mm	12
50+ mm	9
Unknown	13
**T stage**	
T1	0
T2	2
T3	9
T4	16
Tx/Unknown	26
**N stage**	
N0	0
N1	8
N2	12
Nx	100
**Radiotherapy performed**	
No	9
Yes	0
**Chemotherapy performed**	
No	0
Yes	7
**CEA pretreatment**	
Borderline	2
Positive	12
Negative	0
Unknown	4

## Discussion

A major clinical feature of young-onset CRC was frequent involvement of regional lymph nodes and distant organ metastasis, which accounted for most of the deaths (11). Due to the heterogeneity, it is difficult to distinguish sporadic from the hereditary forms of CRC, especially in young-onset CRC patients (12). This is particularly crucial to understand the pathological characteristics of young-onset CRC. However, there was still no research concentrating on the DM of CRC adult cases below 50 years old. Whether DM occurs will directly affect the surgical efficacy and final prognosis of patients. The combination of surgical resection and other systemic treatment can significantly improve the prognosis of CRC patients with distant liver metastases (13, 14). Thus, we successfully explored the risk factors of young-onset CRC patients and constructed a model for predicting DM based on specific pathologic tumor signatures.

In this work, we found that pretreatment CEA positive was an independent risk factor of DM for early stage young-onset CRC patients, which could provide an important clue of DM for clinicians. CEA was a cell surface glycoprotein overexpressed in normal mucosal cells. In fact, previous studies had found that CEA was a strong predictor closely correlated with DM in CRC. Pakdel et al. discovered that preoperative serum CEA concentration in CRC patients was higher in patients with DM than those without DM (15). Guo et al. found N2 stage, positive CEA, and tumor size over 30 mm were predictors of DM in T1 colorectal cancer (16). Liu and his colleagues also discovered that CEA was a risk factor of preoperative synchronous DM in rectal cancer (17). A large sample cohort study found CEA over 6, T4 stage, and N2 stage could be utilized to DM in rectal cancer (18). CEA-targeted nanoparticle therapy was also considered a potential treatment for CRC (19). In addition, CEA was also incorporated in the prediction model of DM of advanced CRC. Consequently, this study emphasized that dynamic monitoring of CEA level postoperative might be an important means of DM in young-onset CRC patients.

Additionally, our study showed that tumor size, undifferentiated carcinoma, tumor grades of T4 stage and N2 stage, treatment without radiation, treated with chemotherapy, and pretreatment CEA positive were associated with DM after surgery in young-onset patients with advanced CRC. Gaitanidis et al. found that factors such as age, sex, race, tumor location, tumor grade, primary tumor size, CEA levels, perineural invasion, T stage, N stage, liver, and lung metastasis were predictors for synchronous DM in rectal cancer (20). Our model also included factors such as tumor grade, tumor size, and pretreatment CEA, which were consistent with previous study. Whereas, demographic features were not identified as risk factors of DM in this study. These risk factors were not only related to DM but also linked with poor OS (21, 22).

Our study demonstrated that larger tumor size was an independent risk factor of DM. Previous study found that tumor size over 20 mm was an independent risk factor of CSS and DM in patients with neuroendocrine tumors (23). The preliminary analysis of Huang et al. showed that the CSS of patients with tumors ≤4.0, 4.0–7.0, and ≥7.0 cm increased continuously for 5 years (24). The results of these studies might be originated from different groupings. More attention should be paid to those with a tumor size larger than 10 mm since they were more likely to develop DM. Our study showed that the degree of carcinoma differentiation was also associated with DM of CRC, which was in line with previous researches (25, 26). Interestingly, tumor location was found more in the rectum than right colon in advanced stage young-onset patients. However, after being adjusted by other factors, it was not included in our prediction model finally. We could not completely deny the clinical value of tumor location in young-onset CRC as many studies have reported that it was one of pivotal signatures in CRC patients (27).

Although the effect of chemotherapy on CRC has been widely studied, the clinical benefits still remained controversial (28). A meta-analysis concluded that postoperative adjuvant chemotherapy could not improve OS, disease-free survival or distant recurrence of rectal cancer (29). Nevertheless, chemotherapy was still regarded as one of the most important palliative cures for CRC patients, which had been accepted as the standard treatment for patients with locally advanced rectal cancer all over the world (10, 30). Most of the studies supported that chemotherapy could improve the prognosis of patients, which could explain why DM patients often received chemotherapy. As for radiotherapy, a study demonstrated that radiotherapy combined with surgery were helpful for patients with local recurrence of rectal cancer (31). Chemotherapy, radiotherapy, and molecular-targeted drug therapy remained the mainstay of treatment for advanced CRC (32). In recent years, researches have detected that chemotherapy with pelvic radiotherapy was more recommended to improve the prognosis of patients with DM (33). More studies were needed to investigate the potential of system treatment for advanced CRC, especially in young-onset CRC patients.

Nomogram was an effective and excellent predictive tool which had a wide range of applications in various studies. Huang and his colleagues created a radiomics nomogram of preoperative lymph node metastasis in patients with CRC to calculate the individualized risk of lymph node metastasis (34). This method was also broadly applied to other carcinomas such as lung (35, 36), esophageal cancer (36), early gastric cancer (37), and so on. Due to limited cohorts, most of the studies could not extend the external verification of this risk model. Therefore, they were unable to evaluate the goodness of fit of the model. In this study, internal and external crossvalidation both showed our model was workable. The AUC, calibration curve, and DCA could better ensure the excellent performance of our model in terms of accuracy, consistency, and clinical applicability. Three groups at low risk, medium risk, and high risk were identified to be associated with survival prognosis. All of the results indicated that our model was reliable and could provide further clues for clinical physicians. Therefore, closely monitoring DM should be considered for young-onset CRC patients with larger tumor size, undifferentiated carcinoma, tumor grades of T4 or N2 stage, treatment without radiation or treatment with chemotherapy after surgery, and pretreatment CEA positive.

Nevertheless, limitations still remained in our study. Although the SEER database provided us with vast data to establish the model, the sample size of the validation cohort was relatively small. Thus, the model required further validation with data from multicenter. Additionally, the lack of data in the SEER database would also have a certain impact on the accuracy of the model. Finally, relatively small number of M1 patients in the early stage cohort might lead to underestimation of the effect of other variables. Further analysis of early stage for young-onset CRC patients could also make sense.

In conclusion, our research successfully identified a number of independent risk factors of DM in different stages and created a predictive nomogram to predict DM in advanced stage for CRC patients younger than 50 years old. Internal verification and external verification of the model both demonstrated good predictive performance. Thus, they can assist clinicians in following disease progression and help tailor therapy strategy accordingly.

## Data Availability Statement

Most of the data made available were derived from SEER-database (https://seer.cancer.gov/). Data of validation set that support the findings of this study are available from the corresponding author upon reasonable request.

## Ethics Statement

The studies involving human participants were reviewed and approved by the ethics committee at Zhongnan Hospital of Wuhan University (number: 2020074). Written informed consent for participation was not required for this study in accordance with the national legislation and the institutional requirements. Written informed consent was not obtained from the individual(s) for the publication of any potentially identifiable images or data included in this article.

## Author Contributions

JC, Y-JL, and QW designed the research and drafted the full manuscript together, including analyzed and interpreted data. J-LM and KH collected the validation data from Zhongnan Hospital of Wuhan University. J-HF and FH revised the manuscript. M-LL participated in data collation and analysis. JL was engaged in the supervision and guidance of the whole research. All authors read and approved the final manuscript.

## Funding

This work was supported by Hubei central government guides local science and technology development special project (number 2020ZYYD012) and Joint Funds Project of Health Commission of Hubei Province (number WJ2019H082).

## Conflict of Interest

The authors declare that the research was conducted in the absence of any commercial or financial relationships that could be construed as a potential conflict of interest.

## Publisher’s Note

All claims expressed in this article are solely those of the authors and do not necessarily represent those of their affiliated organizations, or those of the publisher, the editors and the reviewers. Any product that may be evaluated in this article, or claim that may be made by its manufacturer, is not guaranteed or endorsed by the publisher.

## References

[1] Sung H Ferlay J Siegel RL Laversanne M Soerjomataram I Jemal A. Global Cancer Statistics 2020: GLOBOCAN Estimates of Incidence and Mortality Worldwide for 36 Cancers in 185 Countries. CA Cancer J Clin (2021) 71(3):209–49. doi: 10.3322/caac.21660 33538338

[2] Chambers AC Dixon SW White P Williams AC Thomas MG Messenger DE. Demographic Trends in the Incidence of Young-Onset Colorectal Cancer: A Population-Based Study. Demographic Trends in the Incidence of Young-Onset Colorectal Cancer: A Population-Based Study. Br J Surg (2020) 107(5):595–605. doi: 10.1002/bjs.11486 32149386 PMC7155067

[3] Burnett-Hartman AN Lee JK Demb J Gupta S. An Update on the Epidemiology, Molecular Characterization, Diagnosis, and Screening Strategies for Early-Onset Colorectal Cancer. Gastroenterology (2021) 160(4):1041–9. doi: 10.1053/j.gastro.2020.12.068 PMC8273929 33417940

[4] Stoffel EM Murphy CC. Epidemiology and Mechanisms of the Increasing Incidence of Colon and Rectal Cancers in Young Adults. Gastroenterology (2020) 158(2):341–53. doi: 10.1053/j.gastro.2019.07.055 PMC6957715 31394082

[5] Willauer AN Liu Y Pereira AAL Lam M Morris JS Raghav KPS. Clinical and Molecular Characterization of Early-Onset Colorectal Cancer. Cancer (2019) 125(12):2002–10. doi: 10.1002/cncr.31994 PMC6583775 30854646

[6] van der Geest LG Lam-Boer J Koopman M Verhoef C Elferink MA de Wilt JH. Nationwide Trends in Incidence, Treatment and Survival of Colorectal Cancer Patients With Synchronous Metastases. Clin Exp Metastasis (2015) 32(5):457–65. doi: 10.1007/s10585-015-9719-0 25899064

[7] Filip S Vymetalkova V. Distant Metastasis in Colorectal Cancer Patients-Do We Have New Predicting Clinicopathological and Molecular Biomarkers? A Comprehensive Review. Int J Mol Sci (2020) 21(15):5255. doi: 10.3390/ijms21155255 PMC7432613 32722130

[8] Boardman LA Vilar E You YN Samadder J. AGA Clinical Practice Update on Young Adult-Onset Colorectal Cancer Diagnosis and Management: Expert Review. Clin Gastroenterol Hepatol (2020) 18(11):2415–24. doi: 10.1016/j.cgh.2020.05.058 32525015

[9] Benson AB Venook AP Al-Hawary MM Cederquist L Chen YJ Ciombor KK. NCCN Guidelines Insights: Colon Cancer, Version 2.2018. J Natl Compr Canc Netw (2018) 16(4):359–69. doi: 10.6004/jnccn.2018.0021 PMC10184502 29632055

[10] Benson AB Venook AP Al-Hawary MM Cederquist L Chen YJ Ciombor KK. Rectal Cancer, Version 2.2018, NCCN Clinical Practice Guidelines in Oncology. J Natl Compr Canc Netw (2018) 16(7):874–901. doi: 10.6004/jnccn.2018.0061 30006429 PMC10203817

[11] Kasi PM Shahjehan F Cochuyt JJ Li Z Colibaseanu DT Merchea A. Rising Proportion of Young Individuals With Rectal and Colon Cancer. Clin Colorectal Cancer (2019) 18(1):e87–95. doi: 10.1016/j.clcc.2018.10.002 30420120

[12] Campos FG. Colorectal Cancer in Young Adults: A Difficult Challenge. World J Gastroenterol (2017) 23(28):5041–4. doi: 10.3748/wjg.v23.i28.5041 PMC5537173 28811701

[13] Akgül Ö Çetinkaya E Ersöz Ş Tez M. Role of Surgery in Colorectal Cancer Liver Metastases. World J Gastroenterol (2014) 20(20):6113–22. doi: 10.3748/wjg.v20.i20.6113 PMC4033450 24876733

[14] McNally SJ Parks RW. Surgery for Colorectal Liver Metastases. Dig Surg (2013) 30(4-6):337–47. doi: 10.1159/000351442 24051581

[15] Pakdel A Malekzadeh M Naghibalhossaini F. The Association Between Preoperative Serum CEA Concentrations and Synchronous Liver Metastasis in Colorectal Cancer Patients. Cancer Biomark (2016) 16(2):245–52. doi: 10.3233/cbm-150561 PMC13016458 26756614

[16] Guo K Feng Y. Risk Factors and Predictors of Lymph Nodes Metastasis and Distant Metastasis in Newly Diagnosed T1 Colorectal Cancer. Cancer Med (2020) 9(14):5095–113. doi: 10.1002/cam4.3114 PMC7367623 32469151

[17] Liu H Zhang C Wang L Luo R Li J Zheng H. MRI Radiomics Analysis for Predicting Preoperative Synchronous Distant Metastasis in Patients With Rectal Cancer. Eur Radiol (2019) 29(8):4418–26. doi: 10.1007/s00330-018-5802-7 30413955

[18] Peng J Ding Y Tu S Shi D Sun L Li X. Prognostic Nomograms for Predicting Survival and Distant Metastases in Locally Advanced Rectal Cancers. PloS One (2014) 9(8):e106344. doi: 10.1371/journal.pone.0106344 25171093 PMC4149564

[19] Sousa AR Oliveira MJ Sarmento B. Impact of CEA-Targeting Nanoparticles for Drug Delivery in Colorectal Cancer. J Pharmacol Exp Ther (2019) 370(3):657–70. doi: 10.1124/jpet.118.254441 30670480

[20] Gaitanidis A Alevizakos M Tsaroucha A Tsalikidis C Pitiakoudis M. Predictive Nomograms for Synchronous Distant Metastasis in Rectal Cancer. J Gastrointest Surg (2018) 22(7):1268–76. doi: 10.1007/s11605-018-3767-0 29663304

[21] Li M Zhu YZ Zhang YC Yue YF Yu HP Song B. Radiomics of Rectal Cancer for Predicting Distant Metastasis and Overall Survival. World J Gastroenterol (2020) 26(33):5008–21. doi: 10.3748/wjg.v26.i33.5008 PMC7476170 32952346

[22] Mo S Cai X Zhou Z Li Y Hu X Ma X. Nomograms for Predicting Specific Distant Metastatic Sites and Overall Survival of Colorectal Cancer Patients: A Large Population-Based Real-World Study. Clin Transl Med (2020) 10(1):169–81. doi: 10.1002/ctm2.20 PMC7240852 32508027

[23] Folkert IW Sinnamon AJ Concors SJ Bennett BJ Fraker DL Mahmoud NN. Grade is a Dominant Risk Factor for Metastasis in Patients With Rectal Neuroendocrine Tumors. Ann Surg Oncol (2020) 27(3):855–63. doi: 10.1245/s10434-019-07848-0 31701298

[24] Huang B Feng Y Mo SB Cai SJ Huang LY. Smaller Tumor Size is Associated With Poor Survival in T4b Colon Cancer. World J Gastroenterol (2016) 22(29):6726–35. doi: 10.3748/wjg.v22.i29.6726 PMC4970476 27547015

[25] Chou CL Chang SC Lin TC Chen WS Jiang JK Wang HS. Differences in Clinicopathological Characteristics of Colorectal Cancer Between Younger and Elderly Patients: An Analysis of 322 Patients From a Single Institution. Am J Surg (2011) 202(5):574–82. doi: 10.1016/j.amjsurg.2010.10.014 21872205

[26] Yeo H Betel D Abelson JS Zheng XE Yantiss R Shah MA. Early-Onset Colorectal Cancer is Distinct From Traditional Colorectal Cancer. Clin Colorectal Cancer (2017) 16(4):293–299.e6. doi: 10.1016/j.clcc.2017.06.002 29033218

[27] Liu Z Xu Y Xu G Baklaushev VP Chekhonin VP Peltzer K. Nomogram for Predicting Overall Survival in Colorectal Cancer With Distant Metastasis. BMC Gastroenterol (2021) 21(1):103. doi: 10.1186/s12876-021-01692-x 33663400 PMC7934422

[28] Liu Z Meng X Zhang H Li Z Liu J Sun K. Predicting Distant Metastasis and Chemotherapy Benefit in Locally Advanced Rectal Cancer. Nat Commun (2020) 11(1):4308. doi: 10.1038/s41467-020-18162-9 32855399 PMC7452897

[29] Bujko K Glimelius B Valentini V Michalski W Spalek M. Postoperative Chemotherapy in Patients With Rectal Cancer Receiving Preoperative Radio(Chemo)Therapy: A Meta-Analysis of Randomized Trials Comparing Surgery ± a Fluoropyrimidine and Surgery + a Fluoropyrimidine ± Oxaliplatin. Eur J Surg Oncol (2015) 41(6):713–23. doi: 10.1016/j.ejso.2015.03.233 25911110

[30] Woo IS Jung YH. Metronomic Chemotherapy in Metastatic Colorectal Cancer. Cancer Lett (2017) 400:319–24. doi: 10.1016/j.canlet.2017.02.034 28274890

[31] Ma B Gao P Wang H Xu Q Song Y Huang X. What has Preoperative Radio(Chemo)Therapy Brought to Localized Rectal Cancer Patients in Terms of Perioperative and Long-Term Outcomes Over the Past Decades? A Systematic Review and Meta-Analysis Based on 41,121 Patients. Int J Cancer (2017) 141(5):1052–65. doi: 10.1002/ijc.30805 28560805

[32] Foubert F Matysiak-Budnik T Touchefeu Y. Options for Metastatic Colorectal Cancer Beyond the Second Line of Treatment. Dig Liver Dis (2014) 46(2):105–12. doi: 10.1016/j.dld.2013.07.002 23954144

[33] Wang Y Yu X Zhao N Wang J Lin C Izaguirre EW. Definitive Pelvic Radiotherapy and Survival of Patients With Newly Diagnosed Metastatic Anal Cancer. J Natl Compr Canc Netw (2019) 17(1):29–37. doi: 10.6004/jnccn.2018.7085 30659127

[34] Huang YQ Liang CH He L Tian J Liang CS Chen X. Development and Validation of a Radiomics Nomogram for Preoperative Prediction of Lymph Node Metastasis in Colorectal Cancer. J Clin Oncol (2016) 34(18):2157–64. doi: 10.1200/jco.2015.65.9128 27138577

[35] Wang S Yang L Ci B Maclean M Gerber DE Xiao G. Development and Validation of a Nomogram Prognostic Model for SCLC Patients. J Thorac Oncol (2018) 13(9):1338–48. doi: 10.1016/j.jtho.2018.05.037 PMC7678404 29902534

[36] Semenkovich TR Yan Y Subramanian M Meyers BF Kozower BD Nava R. A Clinical Nomogram for Predicting Node-Positive Disease in Esophageal Cancer. Ann Surg (2021) 273(6):e214–21. doi: 10.1097/sla.0000000000003450 PMC6940556 31274650

[37] Kim SM Min BH Ahn JH Jung SH An JY Choi MG. Nomogram to Predict Lymph Node Metastasis in Patients With Early Gastric Cancer: A Useful Clinical Tool to Reduce Gastrectomy After Endoscopic Resection. Endoscopy (2020) 52(6):435–43. doi: 10.1055/a-1117-3059 32162286

